# Supplementary material for: A Benzothiazole-Based Fluorescent Probe for Ratiometric Detection of Al3+ and Its Application in Water Samples and Cell Imaging
Source: Int J Mol Sci. 2019 Nov 28;20(23):5993. doi: 10.3390/ijms20235993 (PMC6929017; doi:10.3390/ijms20235993)
Supplement: Supplementary file 1 [file ijms-20-05993-s001.pdf]

A benzothiazole-based fluorescent probe for ratiometric detection of Al^{3+} and its application in water samples and cell imaging

Zhen-Nan Tian, Ding-Qi Wu, Xue-Jiao Sun, Ting-Ting Liu, Zhi-Yong Xing *

Department of Applied Chemistry, College of Arts and Sciences, Northeast Agricultural University, Harbin 150030, PR China

* Corresponding author

E-mail addresses: zyxing@neau.edu.cn

Table of Contents

Figure S1. The ^{1}H NMR (DMSO-d_6, 600 MHz) spectrum of compound **BHM**.

Figure S2. The ^{13}C NMR (DMSO-d_6, 151 MHz) spectrum of compound **BHM**.

Figure S3. The ESI-MS spectrum of compound **BHM**.

Figure S4. Absorbance intensity of **BHM** (10 μM) as a function of Al^{3+} (0-12 μM) in DMF-H_2O (1/1, v/v).

Figure S5. Fluorescence intensity of **BHM** (10 μM) as a function of Al^{3+} (12-28 μM) in DMF-H_2O (1/1, v/v).

Figure S6 Benesi-Hildebrand plot of **BHM** (10 μM) assuming a 1:1 stoichiometry for association between **BHM** and Al^{3+} in DMF-H_2O (1/1, v/v) solution by fluorescence spectroscopy.

Figure S7 Benesi-Hildebrand plot of **BHM** (10 μM) assuming a 1:1 stoichiometry for association between **BHM** and Al^{3+} in DMF-H_2O (1/1, v/v) solution by absorption spectroscopy.

Figure S8. Fluorescent detection of **BHM** (10 μM) in water samples upon addition of Al^{3+} (recorded at λ=478 nm).

Figure S9 Linearity with respect to Al^{3+} over the concentration range of 15-20 μM in ultrapure water.

Figure S10 Linearity with respect to Al^{3+} over the concentration range of 15-20 μM in Tap water.

Figure S11 Linearity with respect to Al^{3+} over the concentration range of 15-20 μM in Songhua River.

Table S1 Crystal data and structure refinement for **BHM**

Table S2 Fractional Atomic Coordinates (×10^4) and Equivalent Isotropic Displacement Parameters (Å^2×10^3) for **BHM**.

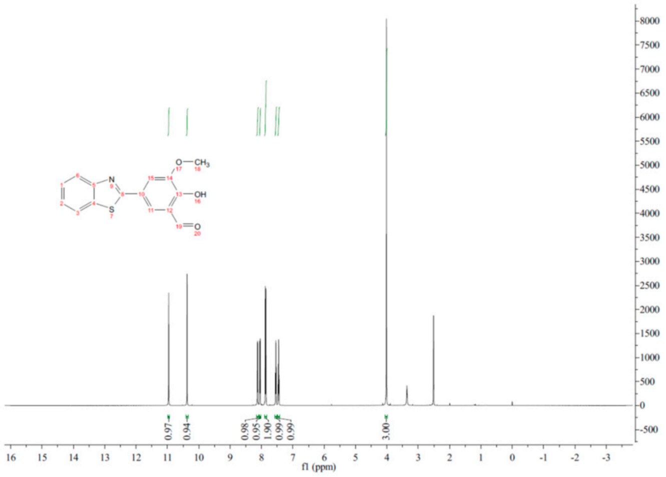

Figure S1. The ^{1}H NMR (DMSO-d$_6$, 600 MHz) spectrum of compound **BHM**.

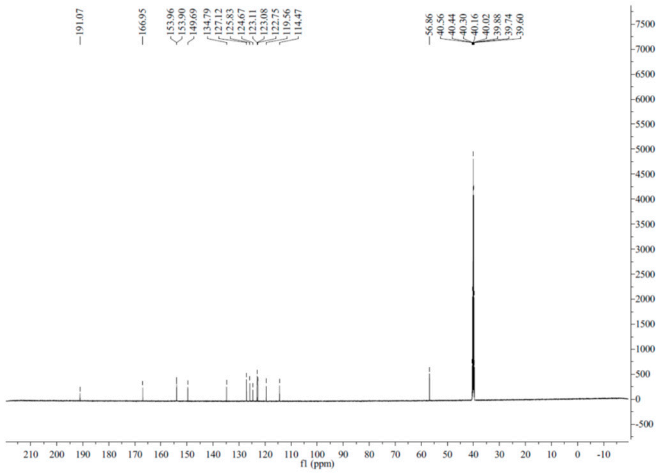

Figure S2. The ^{13}C NMR (DMSO-d$_{6,}$ 151 MHz) spectrum of compound **BHM**.

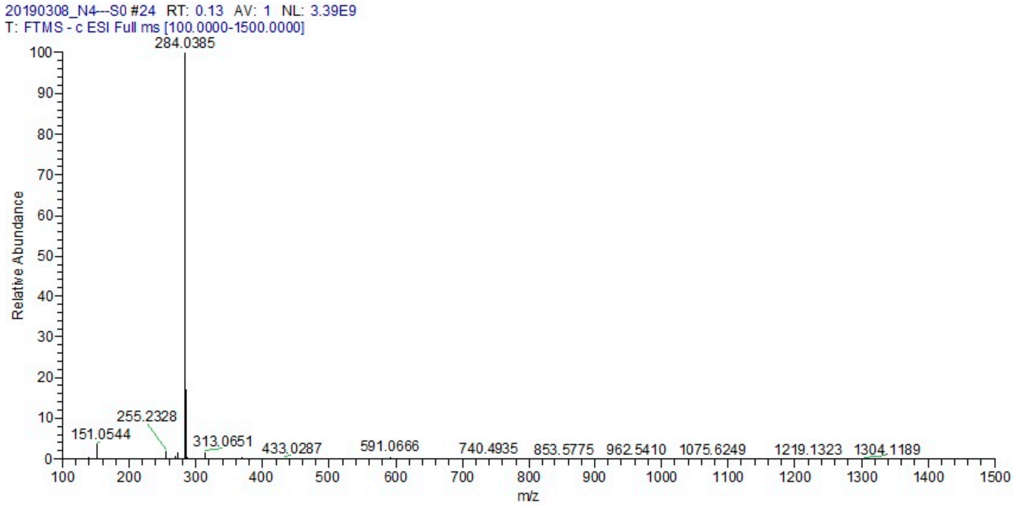

Figure S3. The ESI-MS spectrum of compound **BHM**.

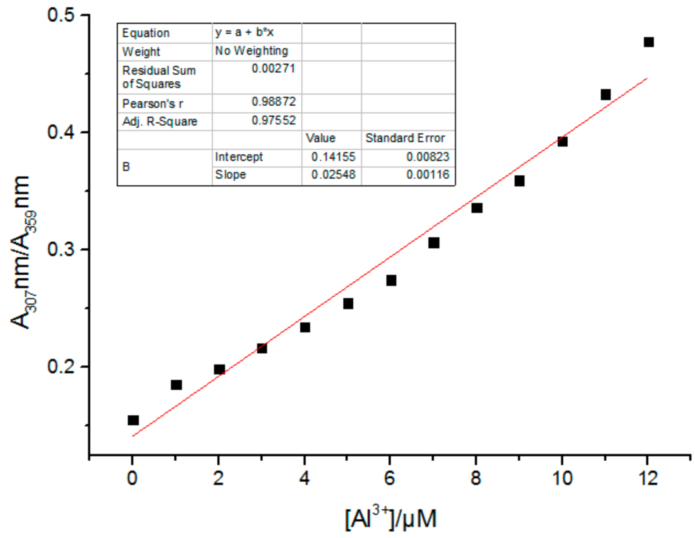

Figure S4. Absorbance intensity of **BHM** (10 μM) as a function of Al^{3+} (0-12 μM) in DMF-H_2O (1/1, v/v).

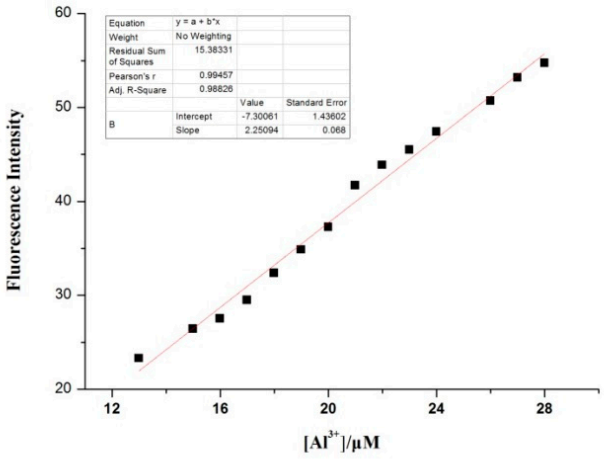

Figure S5. Fluorescence intensity of **BHM** (10 μM) as a function of Al^{3+} (12-28 μM) in DMF-H_2O (1/1, v/v).

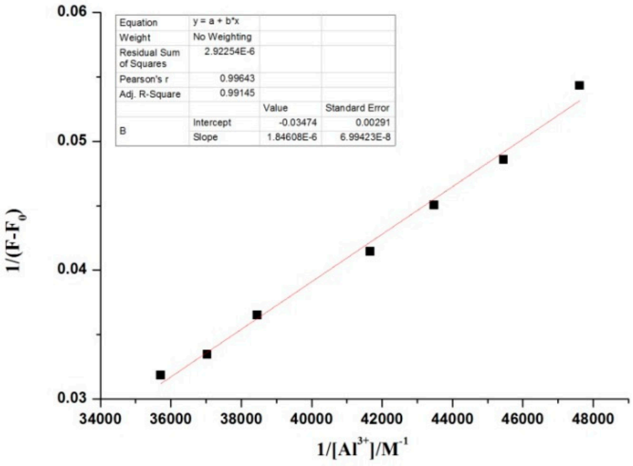

Figure S6 Benesi-Hildebrand plot of **BHM** (10 μM) assuming a 1:1 stoichiometry for association between **BHM** and Al^{3+} in DMF-H_2O (1/1, v/v) solution by fluorescence spectroscopy.

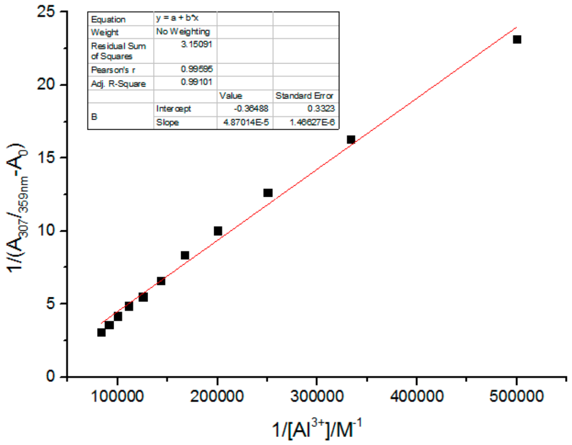

Figure S7 Benesi-Hildebrand plot of **BHM** (10 μM) assuming a 1:1 stoichiometry for association

between **BHM** and Al^{3+} in DMF-H_2O (1/1, v/v) solution by absorption spectroscopy.

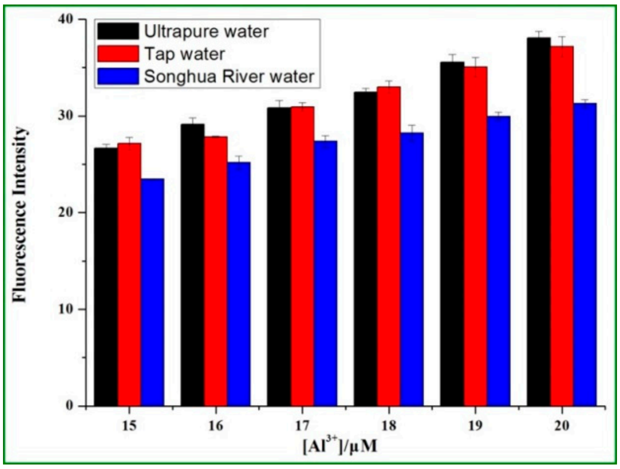

Figure S8. Fluorescent detection of **BHM** (10 μM) in water samples upon addition of Al^{3+} (recorded at λ=478 nm).

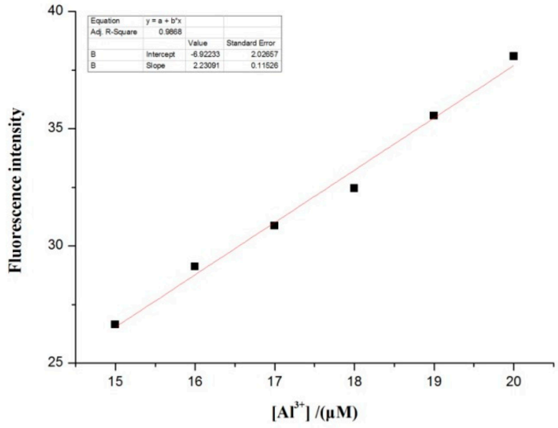

Figure S9 Linearity with respect to Al^{3+} over the concentration range of 15-20 μM in ultrapure water.

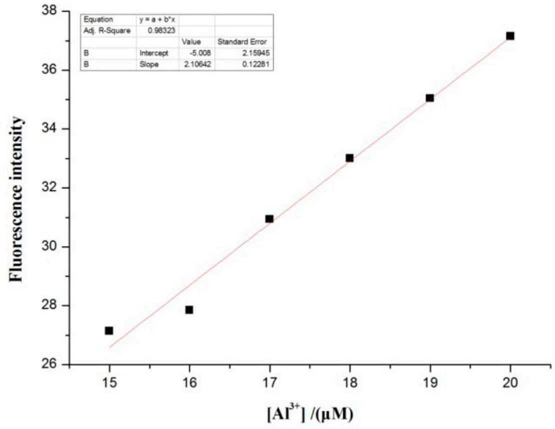

Figure S10 Linearity with respect to Al^{3+} over the concentration range of 15-20 μM in Tap water.

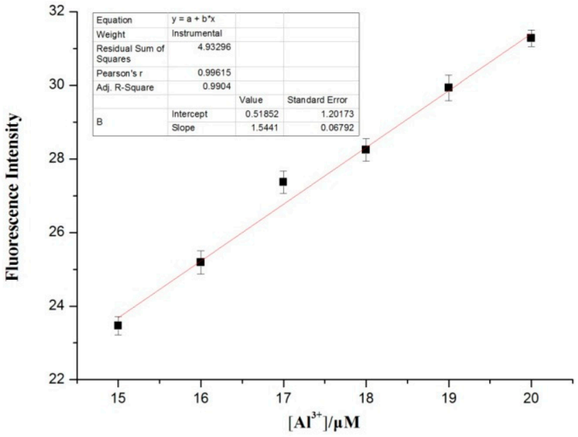

Figure S11 Linearity with respect to Al^{3+} over the concentration range of 15-20 μM in Songhua River.

Table S1 Crystal data and structure refinement for **BHM**

CCDC	1902058	P(g/cm^3)	1.495
Empirical formula	$C_{15}H_{11}NO_3S$	μ/mm^{-1}	0.261
Formula weight	285.31	F(000)	1184.0
Temperature/K	153.15	Crystal size/mm^3	0.24 × 0.23 × 0.2
Crystal system	orthorhombic	Radiation	MoKα (λ = 0.71073)
Space group	Pbcn	2θ range for data collection/°	4.97 to 54.958
a/Å	10.566(2)	Index ranges	$-13 \le h \le 8$; $-16 \le k \le 16$; $-23 \le l \le 15$
b/Å	12.984(3)	Reflections collected	9673
c/Å	18.484(4)	Independent reflections	2896 [R_{int} = 0.0434, R_{sigma} = 0.0409]
α/°	90	Data/restraints/parameters	2896/0/182
β/°	90	Goodness-of-fit on F^2	1.131
γ/°	90	Final R indexes [I>=2σ (I)]	R_1 = 0.0517, wR_2 = 0.1102
Volume/Å^3	2535.7(9)	Final R indexes [all data]	R_1 = 0.0550, wR_2 = 0.1121
Z	8	Largest diff. peak/hole / e Å^{-3}	0.27/-0.40

Table S2 Fractional Atomic Coordinates (×10^4) and Equivalent Isotropic Displacement Parameters (Å^2×10^3) for **BHM**.

**Atom**	***x***	***y***	***z***	**U(eq)**
S1	177.6(5)	3698.2(3)	1257.8(3)	24.93(15)
O3	-3957.6(15)	6334.1(12)	3119.5(8)	36.1(4)
O1	-934.7(15)	8578.8(10)	1688.0(8)	34.1(4)
N1	1160.3(16)	5308.1(12)	637.7(9)	27.1(4)
C15	41(2)	8940.5(16)	1216.8(12)	33.8(5)
C8	-504.1(18)	5754.5(14)	1496.9(10)	23.7(4)
C6	1770.2(18)	4450.2(14)	349.7(11)	24.5(4)
C1	1368.0(19)	3496.6(14)	625.9(10)	24.1(4)
C13	-1469.5(19)	5425.7(15)	1942.9(10)	26.0(4)
O2	-2733.7(15)	7927.4(11)	2540.0(8)	35.4(4)
C9	-300.7(19)	6819.9(15)	1400.2(11)	25.1(4)
